# A SNP-Based High-Density Genetic Map of Leaf and Fruit Related Quantitative Trait Loci in Wolfberry (*Lycium* Linn.)

**DOI:** 10.3389/fpls.2019.00977

**Published:** 2019-08-07

**Authors:** Jianhua Zhao, Yuhui Xu, Haoxia Li, Yue Yin, Wei An, Yanlong Li, Yajun Wang, Yunfang Fan, Ru Wan, Xin Guo, Youlong Cao

**Affiliations:** ^1^National Wolfberry Engineering Research Center, Ningxia Academy of Agriculture and Forestry Sciences, Yinchuan, China; ^2^Biomarker Technology Corporation, Beijing, China; ^3^Desertification Control Research Institute, Ningxia Academy of Agriculture and Forestry Sciences, Yinchuan, China

**Keywords:** *Lycium* L., SLAF-seq, genetic map, leaf and fruit related traits, quantitative trait locus

## Abstract

Wolfberry (*Lycium* Linn. 2*n* = 24) fruit, Gouqizi, is a perennial shrub, traditional food and medicinal plant resource in China. Leaf and fruit related characteristics are economically important traits that are the focus for genetic improvement, but few studies into the molecular genetics of this crop have been reported to date. Here, an F_1_ population (302 individuals) derived from a cross between “NO.1 Ningqi” (*Lycium barbarum* L.) and “Chinese gouqi” (*Lycium chinese* Mill.) was constructed. We recorded fruit weight, longitude, diameter and index along with leaf length, width and index for three consecutive years from 2015 to 2017. Based on this population and these phenotypic data, we constructed the first high-density genetic map of *Lycium* using specific length amplified fragment sequencing (SLAF-seq) and analyzed quantitative trait loci (QTLs). The map contains 6733 single nucleotide polymorphisms and 12 linkage groups (LG) with a total map distance of 1702.45 cM and an average map distance of 0.253 cM. A total of 55 QTLs were mapped for more than 2 years, of which 18 stable QTLs for fruit index on LG 11, spanning an interval of 73.492–90.945 cM, were detected. *qFI11-15* for fruit index was an impressive QTL with logarithm of odds (LOD) and proportion of variance explained (PEV) values reaching 11.07 and 19.7%, respectively. The QTLs on LG 11 were gathered tightly, having an average interval of less than 1 cM per QTL, suggesting that there might be a cluster region controlling fruit index. Remarkably, *qLI10-2* and *qLI11-2* for leaf index were detectable for 3 years. These results give novel insight into the genetic control of leaf and fruit related traits in *Lycium* and provide robust support for undertaking further positional cloning studies and implementing marker-assisted selection in seedlings.

## Introduction

Wolfberry (*Lycium* Linn. 2*n* = 24) is an important plant resource for both food and medicine in China and has been cultivated for more than 500 years in Ningxia (Yao et al., 2018a). The shrub is a perennial, deciduous member of the Solanaceae family. Dried wolfberry fruit, Gouqizi, has been used for centuries in traditional herbal medicines and nourishing tonics (National Pharmacopoeia Committee, 2015; Chen et al., 2018). This valuable fruit is exported world-wide to consumers who have become increasingly accustomed to its taste and efficacy (Li and Jiang, 2014). Recently, Gouqizi consumption has risen steadily following the publication of medical research into the health benefits of ingesting wolfberry fruit (Amagase et al., 2009; Hsu et al., 2017; Yao et al., 2018b; Tian et al., 2019). The leaves of wolfberry can be used in cooking and dried to produce tea (Dong et al., 2009). Wolfberry shrubs are fast-growing with well-developed deep root systems and extensive adaptability to drought and cold. It is a pioneer species mainly planted in salty and alkaline areas in Northwest China (Liu Y. et al., 2018; Zhao et al., 2018).

Developing cultivars that bear fruit with characteristics that appeal to consumers will help to strengthen the wolfberry production industry. However, there has been a lack of genetic studies into the yield and quality of this crop. Current research is, in the main, limited to phenotypic research. A basic system for evaluating wolfberry fruit was established by An et al. (2007). Since then, a more comprehensive assessment system for fresh fruit has been developed and this was updated in 2017. Additionally, a useful collection of germplasm has been obtained (Shu et al., 2017; Zhao et al., 2018). Physiological research into the accumulation of sugars and acids in the plants and the response to salt stress in the shrub has also been carried out (Zhao et al., 2015, 2017). Recently, the identification of microRNAs present during anther development has provided valuable information about the molecular mechanism of male sterility in *Lycium barbarum* L. (Shi et al., 2018). Currently, there remains a large gap between research into the molecular biology of wolfberry and that for other plants, particularly model species. Moreover, it usually takes 2 years from germination to flowering for wolfberry, hybridization or backcrossing plus multiple rounds of phenotypic selection usually takes about 10 years. Therefore, improving the breeding efficiency of wolfberry is a huge challenge.

Genetic map based on F_1_ population for perennial fruit crops is a robust tool to identify the linkage between horticultural traits and molecular markers (Harel-Beja et al., 2015; Zhu et al., 2015). Moreover, with the rapid popularization of next-generation sequencing (NGS) technology, genome-wide single nucleotide polymorphisms (SNPs) have been used extensively to identify quantitative trait loci (QTLs) through creation of high density genetic maps or by means of bulk segregation analysis (Wang et al., 2012). For species that don’t have a reference genome, restriction enzyme digestion-based methods are effective for finding genome-wide SNPs. These techniques typically include restriction site-associated sequencing (RAD-seq), genotyping-by–sequencing and specific length amplified fragment sequencing (SLAF-seq) (Sun et al., 2013). Among these methods, SLAF-seq has proven to be an efficient technique for large-scale *de novo* SNP discovery and genotyping using high-throughput sequencing platforms. Moreover, it provides a high-resolution strategy for large-scale genotyping and genetic map generation that is applicable to a wide range of species and populations. Specifically, SLAF-based genetic maps have been reported in different perennial woody plants through the study of F_1_ populations (Zhu et al., 2015; Luo et al., 2016; Zhao et al., 2016; Fang et al., 2018; Lu et al., 2018).

Leaf shape is vital to yield performance in plants (Vlad et al., 2014; Teichmann and Muhr, 2015; Liu et al., 2016; Runions et al., 2017). Leaves and fruits are important components of the source-sink system in plants; the coordination of leaf and fruit shape is important to improving yield (Sonnewald and Fernie, 2018). In wolfberry, leaves and fruits differ amongst diverse germplasm resources. The leaves of wolfberry are needle-shaped, but bred cultivars have longer leaves and bear larger fruits, while the leaves of wild-type plants are predominantly narrow and the fruits are smaller (Chen et al., 2018; Zhao et al., 2018).

The purpose of this study was to construct a high density genetic map and determine the genetic characteristics of leaf- and fruit-related traits using NGS technology and population genetics methods, and next to enable the identification of the corresponding QTLs and potential molecular markers tightly linked with good traits.

## Materials and Methods

### Mapping Population Construction and Planting

**FIGURE 1:**
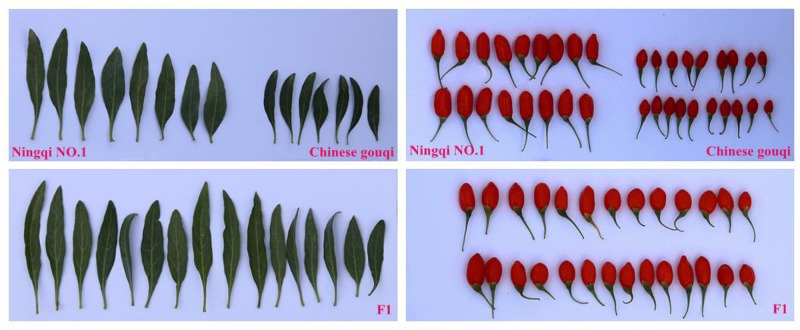
Leaf and fruit growth performance of Ningqi NO.1, Chinese gouqi and F_1_ individuals.

A hybrid wolfberry population derived from a cross between “Ningqi NO.1” (*Lycium barbarum* L.) and “Chinese gouqi” (*Lycium chinese* Mill.) was generated in August 2014. The female parent “Ningqi NO.1” is an artificial breeding cultivar and the major wolfberry cultivar in north-west China. Its fruit is bright red and elliptical, while the leaf is lanceolate. The male parent “Chinese gouqi” is wild-type wolfberry. Its fruit is dark red, smaller and oblate; it is very tolerant to drought (Figure 1). The seeds of the hybrid F_1_ and the two parents were collected and sown in the Ningxia Academy of Agriculture and Forestry Sciences, National Wolfberry Germplasm Resources Garden (38° 380′ N, 106°9′ E), Yinchuan City, Hui Autonomous Region, Ningxia, China, in May of 2015. A total of 500 F_1_ individuals were produced, of which 302 randomly selected individuals were adopted for the mapping population. Water and fertilizer management was the same as that for the production field. Weeds were managed manually.

### Phenotyping

For three consecutive years, the phenotypes of leaf and fruit related traits were recorded. Leaf length (LL) is the maximum distance between the leaf base and tip, leaf width (LW) is the widest distance across the leaf, single fruit weight (FW) is the weight of one mature fruit, fruit longitude (FL) is the maximum distance between fruits top to bottom, and fruit diameter (FD) is the widest distance across a fruit. LL, LW, FW, FL, and FD were measured according to the methods of Shi et al. (2012). LL, LW, FL, and FD were measured using vernier calipers, and FW was acquired using an electronic balance (SE602F, Ohaus, NJ, United States). LI and FI were calculated according to the following formulas: (i) LI = LL/LW; and (ii) FI = FL/FD. LL, LW and LI were recorded for three consecutive years from 2015 to 2017. FW, FL, FD and FI were recorded from 2016 to 2017. Phenotyping assays were conducted each July when summer fruits were ripe.

Since the growth of individual shrubs varied year on year, traits were measured in replicate. In 2015, 15 randomly selected leaves were evaluated for the four leaf-related traits. In 2016 and 2017, 20–30 replicate measurements were made of all seven traits. To establish more precise results, we ranked all measured values for each trait and individual and removed the maximum and the minimum values, leaving nine valid repeated measurements for 2015 and ten for both 2016 and 2017. Complex variance analysis, variance analysis and correlation analysis were carried out using the SPSS V17.0 software package (SPSS Inc., Chicago, IL, United States). Healthy young leaves (–2 g) were harvested separately from both parents and each F_1_ plant in July 2015, immediately soaked in liquid nitrogen and stored at −80°C in a freezer (Thermo Scientific, Waltham, MA, United States). Genomic DNA was extracted using a Trizol Kit (Tiangen Biotech, Beijing, China). DNA concentration was measured using a NanoDrop spectrophotometer (ND2000, Thermo Fisher Scientific) and DNA quality was monitored by electrophoresis on 0.85% agarose gels.

### Specific Length Amplified Fragment Library Generation High-Throughput Sequencing

The prepared genomic DNA samples from all the F_1_ individuals, “Ningqi NO.1” and “Chinese gouqi” were digested using a combination of *Rsa*I and *HinC*II (New England Biolabs, NEB, United States). Nucleotide (A) and duplex tag-labeled sequencing adapters were added to the digested products according to the method established by Luo et al. (2016). Eight cycles of traditional polymerase chain reaction (PCR) were performed with forward primer: 5′-AATGATACGGCGACCACCGA-3′ and reverse primer: 5′-CAAGCAGAAGACGGCATACG-3′) (Life Technologies, Gaithersburg, MD, United States), restriction ligation DNA samples, dNTPs and Q5^R^ High-Fidelity DNA polymerase. Restriction-ligation-PCR products (364–411 bp) were then excised and purified using a QIAquick gel extraction kit (Qiagen, Hilden, Germany). Gel-purified products from each sample were pooled, loaded on three lanes and subjected to cBot cluster generation and sequencing using an Illumina HiSeq platform for 125 pair-end sequencing (Illumina, Inc., San Diego, CA, United States) according to the manufacturer’s instructions.

### Single Nucleotide Polymorphism Development and Genotyping

Raw sequencing data were quality controlled by an in-house Perl script developed by Biomarker Technologies Co., Ltd., (Beijing, China). According to quality control criteria, reads for which >50% of bases had *Q* values ≤ 10 or ambiguous sequence content (“N”) of >10% were removed. The remaining reads were sorted by individual using duplex barcode sequences before trimming the barcodes. The subsequent clean sequence reads were uploaded onto the sequence read archive of the NCBI database (Accession number: PRJNA513212). BLAT software was used to group SLAF reads with parameter similarity = 90% and mean scores = 45 (Kent, 2002). A slightly modified SNP calling pipeline developed by Sun et al. (2013) was used. SLAF-tags with more than three SNPs were filtered out and individual loci with more than four genotypes were excluded because, for a diploid species, a single SLAF locus cannot capture more than four genotypes. SNPs with sequencing depths of more than fourfold in each parent were reserved and classified into eight segregation patterns (aa × bb, ab × cd, ef × eg, hk × hk, lm × ll, nn × np, ab × cc and cc × ab). All patterns except aa × bb were selected to construct a high-density genetic map for a cross pollinator population.

### Genetic Linkage Map Construction and QTL Mapping

First, we carried out two-step SNP marker filtering to ensure that all eligible SNP markers were reserved for subsequent genetic map construction. Filtering was based on integrity (≤75%) and highly significant segregation distortion (SD; *P* < 0.01) as calculated using the Chi-square test. Secondly, SNP markers were arranged in linkage groups (LGs) based on the values of pairwise modified logarithm of odds (MLOD). Markers with MLOD scores >9 and recombination rate <0.4 were grouped into a single LG. SMOOTH algorithms (Van Ooijen, 2004) were used to correct genotypes and imputation. HighMap software was applied to order the SLAF markers within LGs (Liu et al., 2014). Map distances were estimated using the Kosambi mapping function (Kosambi, 1943).

The mean values for each trait and individual for each year were used as the final phenotypes for QTL mapping. MapQTL V5.0 was used to conduct QTL analyses (Van Ooijen, 2004) using the interval mapping model. The threshold LOD value for significance was 2.5. The percentage of phenotype variance explained by its corresponding QTL (Expl. %) was calculated based on the population variance within the segregating population. QTL naming was carried out according to McCouch et al. (1997).

## Results

### Leaf and Fruit Trait Variation Analyses

All leaf related agronomic traits were evaluated for three continuous years from 2015 to 2017 and fruit related traits were evaluated for two continuous years from 2016 to 2017. Complex variance analysis demonstrated that significant differences (*P* < 0.05 or *P* < 0.01) existed in all seven traits (LL, LW, LI, FW, FL, FD, and FI) in different years and F_1_ offspring, indicating that these traits can easily be affected by changing environmental conditions and abundant variation exists (Table 1). As shown in Table 2 and Supplementary Figure S1, the seven traits showed diverse variation in 302 offspring with coefficient of variation values ranging from 9 for FL in 2016 to 30 for FW in 2017. Values from Kolmogorov-Smirnov tests ranged from 0.8945 to 0.9022, indicating that all traits belonged to a positively normal distribution. Correlation analysis (Table 3) showed that significant or extreme positive correlations (*P* < 0.05 or *P* < 0.01) existed in coupled comparisons between FL and FW, FD and FW, FD and FL, LL and FI, LW and FW, LW and FL, LI and FI, and LI and LL, while FI and LW, LI and FW, and LI and LW pairs showed significant or extreme negative association (*P* < 0.05 or *P* < 0.01).

**TABLE 1:** Complex variance analysis of leaf- and fruit-related traits.

**Source**	**Leaf length (LL)**			**Leaf width (LW)**			**Leaf index (LI)**			**Fruit weight (FW)**			**Fruit longitude (FL)**			**Fruit diameter (FD)**			**Fruit index (FI)**		
	
	**DF**	**MS**	***F* value**	**DF**	**MS**	***F* value**	**DF**	**MS**	***F* value**	**DF**	**MS**	***F* value**	**DF**	**MS**	***F* value**	**DF**	**MS**	***F* value**	**DF**	**MS**	***F* value**
Repetition	8	493.90	7.72^∗^	8	609.26	118.91^∗∗^	8	93.91	174.55	8	84.99	49.69^∗∗^	8	2812.62	327.85^∗∗^	8	338.75	234.96^∗∗^	8	9.75	188.98^∗∗^
Year	2	2261.70	35.25^∗∗^	2	4533.59	878.16^∗∗^	2	718.32	1325.96^∗∗^	1	96.21	53.94^∗∗^	1	22340.55	2600.18^∗∗^	1	2671.95	1848.69^∗∗^	1	76.33	1475.26^∗∗^
Offspring	301	745.73	54.04^∗∗^	301	56.97	50.25^∗∗^	301	7.10	50.80^∗∗^	301	2.23	1.31^∗^	301	107.97	119.71 ^∗^	301	15.77	55.73	301	0.70	80.10^∗∗^

*^∗^ and ^∗∗^, 0.05 and 0.01 significance by one-way ANOVA, respectively. MS, mean square; DF, degrees of freedom.*

**TABLE 2:** The characteristics of leaf- and fruit-related traits of F_1_ individuals from 2015 to 2017.

**Trait**		**Year**	**Mean ± SD**	**Maximum**	**Minimum**	**Skewness**	**Kurtosis**	**Coefficient of variance (%)**	**Variance**	***P* value**
Leaf traits^a^	Leaf length (LL)	2015	42.96 ± 6.68	64.19	27.84	0.35	–0.15	16	44.62	0.18
		2016	38.00 ± 6.39	54.12	24.24	0.21	–4.60	17	40.81	0.79
		2017	39.15 ± 7.67	60.50	21.15	0.14	–0.43	20	58.72	0.61
	Leaf width (LW)	2015	8.24 ± 1.65	14.84	4.24	0.47	0.17	20	2.72	0.90
		2016	8.48 ± 1.46	13.04	5.63	0.44	0.16	17	2.12	0.90
		2017	10.13 ± 2.28	19.20	4.8	0.76	0.86	23	5.20	0.90
	Leaf index (LI)	2015	5.34 ± 0.87	8.49	3.26	0.37	0.75	16	0.75	0.90
		2016	4.57 ± 0.76	7.33	3.2	0.94	1.02	17	0.58	0.90
		2017	4.01 ± 1.10	13.99	1.17	0.86	1.03	25.1	1.01	0.90
Fruit traits^b^	Single fruit	2016	0.46 ± 0.11	0.84	0.24	0.24	0.90	24	0.01	0.90
	weight (FW)	2017	0.71 ± 0.21	1.43	0.27	0.05	0.21	30	0.05	0.90
	Fruit longitude (FL)	2016	8.43 ± 0.72	10.78	6.87	0.52	0.40	9	052	0.90
		2017	9.46 ± 1.19	12.28	6.18	–0.64	0.39	13	1.41	0.90
	Fruit diameter (FD)	2016	13.26 ± 1.73	21.97	9.86	1.00	2.41	13	3.00	0.90
		2017	16.38 ± 3.06	25.09	7.79	–0.57	0.51	19	9.37	0.90
	Fruit Index (FI)	2016	4.57 ± 0.76	7.33	3.2	0.86	0.94	17	0.58	0.90
		2017	3.94 ± 0.58	6.12	2.65	1.03	1.00	15	0.34	0.90

*^*a*^Leaf data were collected from 2015 to 2017. ^*b*^Fruit data were collected from 2016 to 2017. *P* > 0.05 indicates that it is normally distributed.*

**TABLE 3:** The correlation between leaf- and fruit-related traits.

	**Fruit weight (FW)**	**Fruit longitude (FL)**	**Fruit diameter (FD)**	**Fruit index (FI)**	**Leaf length (LL)**	**Leaf width (LW)**	**Leaf index (LI)**
Fruit weight (FW)	1						
Fruit longitude (FL)	0.92^∗∗^	1					
Fruit diameter (FD)	0.84^∗∗^	0.71^∗∗^	1				
Fruit index (FI)	–0.15	–0.11	–0.10	1			
Leaf length (LL)	0.03	0.07	0.001	0.28^∗∗^	1		
Leaf width (LW)	0.26^∗^	0.26^∗^	0.162	–0.46^∗∗^	0.55	1	
Leaf index (LI)	−0.21^∗^	0.193	–0.12	0.82^∗∗^	0.244^∗^	−0.64^∗∗^	1

*^∗^ and ^∗∗^ represent significant correlation at *P* < 0.05 and *P* < 0.01, respectively.*

### Specific Length Amplified Fragment Sequencing and Genotyping

High-throughput SLAF sequencing generated a total of 1,670 million clean pair-end reads with 24.43 and 14.34 million in the female and male parents, respectively. Approximately 5.40 million reads per individual were generated, of which the average Q30 was 95.15%, indicating that high-quality source data was generated. The average guanine-cytosine content was 39.67% and the fluctuation was less than 1.5% among offspring and parents. Correspondingly, a total of 1,078,383 SLAFs were detected with 377,632 in the female parent and 342,348 in the male parent, respectively. The average depths were 51.17-fold and 30.61-fold in the female parent and male parent, respectively. The average number of SLAFs for all 302 offspring was 201,073 with an average depth of 21.77-fold (Table 4 and Supplementary Table S1).

**TABLE 4:** Data generated during sequencing of the SLAF library.

**Samples**	**Number of reads**	**GC Contents (%)**	**Q30 (%)**	**Number of SLAFs**	**Total depth**	**Average depth**
Male parent	14,340,441	40.56	95.28	377,632	19,324,798	51.17
Female parent	24,437,504	39.63	95.59	342,348	10,478,630	30.61
Offspring (average)	5,402,702	39.67	95.15	201,073	4,448,262	21.77
Total	1,670,393,964	39.67	95.15	1,078,383	–	–

Based on these high-quality data, we identified 672,790 SNPs in total. The numbers of SNPs detected in both the parents and offspring were 167,501 and 102,920 where a parental sequence depth of greater than four was used for segregation pattern classification. Detailed SNP classification information is shown in Table 5. As we had sufficient numbers of SNPs and fewer were classified into patterns ab × cc, cc × ab and ef × eg, SNPs in patterns hk × hk, lm × ll and nn × np were employed for pre-genetic map construction. To ensure the quality of the genetic map, markers with integrity ≤75% and highly significant SD (*P* < 0.01) were filtered out. Finally, a set of 6962 SNPs was used to construct the first high-density genetic map of wolfberry.

**TABLE 5:** Segregation patterns of SLAF markers.

**Filtered step**		**Numbers**
Total Number of SLAFs		1,078,383
Total SNP markers		672,790
Polymorphic SNP markers		167,501
SNP markers with the parental reads sequence depth more than four in the SLAFs		102,920
SNP markers genotypes	aa × bb	17,513
	ab × cc	108
	cc × ab	118
	ef × eg	572
	hk × hk	20,516
	lm × ll	13,237
	nn × np	50,856
	ab × cd	0

### Characteristics of the High-Density Genetic Map

**FIGURE 2:**
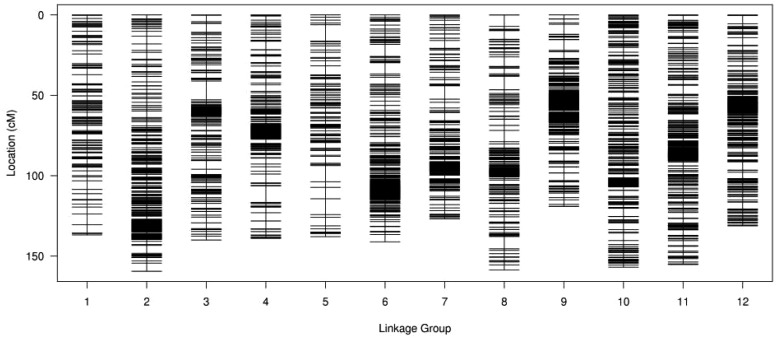
Distribution of SNP markers on 12 LGs of *Lycium*. A black bar indicates a SNP marker, *x*-axis indicates LG and *y*-axis represents genetic distance (cM).

From the final number of 6962 SNP markers, 6,733 were successfully integrated into female and male maps containing 4946 and 2455 SNPs, respectively. The integrated genetic map includes 12 LGs with a total genetic distance of 1702.45 cM and an average map distance of 0.25 cM (Figure 2). The highest number of markers (949) were located in LG11 with an average distance of 0.16 cM and a total genetic distance of 155.25 cM. The lowest number of markers (204) was in LG05 spanning 137.92 cM with an average distance of 0.68 cM. The largest gap stretched across 11.70 cM on LG03. The ratios of genetic distance between adjacent markers less than 5 cM ranged from 97.04 to 100% on LG10 and LG03, respectively. More detailed information, including total marker numbers, total distance, average distance, gaps below 5 cM and the maximum gap, is shown in Supplementary Table S2.

### Quantitative Trait Loci Mapping of Leaf and Fruit Related Traits

Using the interval mapping model in MapQTL V5.0, we mapped a large number of QTLs responsible for the seven traits (data not shown). In this paper, we measured traits for 3 years and found that significant or highly significant differences existed in all seven traits for different years, so we set the LOD threshold = 2.5 and focused our attention on QTLs that were detected steadily, meaning QTLs mapped in more than 2 years. Table 6 and Supplementary Figure S2 show all QTLs that were detected in more than 2 years.

**TABLE 6:** Stable QTLs.

**Trait**	**Year**	**QTL**	**Linkage group**	**Map position**		**Marker number**	**LOD**	**PVE (%)**
				**Start (cM)**	**End (cM)**			
FW	2016/2017	*qFW1*	1	96.964	103.177	4	2.74–4.14	4.7–8.8
	2016/2017	*qFW7*	2	7.878	9.4	2	2.64–2.88	4.9–5.8
	2016/2017	*qFW8-1*	8	111.218	111.367	2	2.51–2.81	4.3–5.4
	2016/2017	*qFW8-2*	8	117.054	117.209	2	2.99–3.06	5.2–5.8
	2016/2017	*qFW10-1*	10	127.423	128.941	1	2.71–3.85	4.6–7.4
	2016/2017	*qFW10-2*	10	133.608	140.357	3	2.64–2.74	4.7–5.2
	2016/2017	*qFW10-3*	10	146.441	147.859	7	2.56–3.09	4.5–6.1
FL	2016/2017	*qFL10*	10	125.297	129.356	6	2.80–3.78	4.8–7.3
	2016/2017	*qFL11-1*	11	72.674	73.702	3	2.95–7.03	5–16.6
	2016/2017	*qFL11-2*	11	82.337	83.89	3	2.51–14.31	4.4–26.7
	2016/2017	*qFL11-3*	11	86.176	86.995	3	2.52–17.24	4.3–30.9
	2016/2017	*qFL11-4*	11	87.186	87.273	4	2.52–15.42	4.3–26.3
	2016/2017	*qFL11-5*	11	87.874	88.019	6	2.50–14.46	4.3–26.9
FD	2016/2017	*qFD8*	8	93.537	94.402	1	2.51–3.19	4.3–10.7
	2016/2017	*qFD10-1*	10	133.608	140.357	4	2.51–3.46	4.5–6.8
	2016/2017	*qFD10-2*	10	146.441	146.903	4	2.7–3.52	4.6–6.7
	2016/2017	*qFD12*	12	65.08	67.33	1	2.62–3.60	5.5–8
FI	2016/2017	*qFI1-1*	1	108.547	108.547	2	2.61–3.7	4.8–7.7
	2016/2017	*qFI1-2*	1	114.807	123.772	5	2.59–3.38	4.5–7.5
	2016/2017	*qFI1-3*	1	135.662	136.861	3	2.56–3.32	5.7–9.0
	2016/2017	*qFI3*	3	85.253	85.367	2	2.54–5.13	4.5–9.8
	2016/2017	*qFI4-1*	4	57.802	57.802	1	2.55–2.56	4.7–5.0
	2016/2017	*qFI4-2*	4	59.872	60.659	10	2.54–3.31	4.5–6.3
	2016/2017	*qFI11-1*	11	73.492	74.349	8	2.64–4.59	4.5–10.5
	2016/2017	*qFI11-2*	11	76.171	78.506	10	2.50–7.07	4.3–13.7
	2016/2017	*qFI11-3*	11	78.734	78.764	1	2.64–6.44	4.6–13.0
	2016/2017	*qFI11-4*	11	79.01	79.045	3	4.85–7.33	8.2–14.6
	2016/2017	*qFI11-5*	11	79.19	79.462	11	4.04–7.52	6.8–13.9
	2016/2017	*qFI11-6*	11	80.021	80.09	2	3.45–7.39	5.9–13.8
	2016/2017	*qFI11-7*	11	80.426	83.667	45	3.42–10.06	5.8–18.4
	2016/2017	*qFI11-8*	11	83.975	84.555	17	2.77–8.86	4.7–16.7
	2016/2017	*qFI11-9*	11	84.691	84.791	6	4.22–6.77	7.1–12.5
	2016/2017	*qFI11-10*	11	84.804	84.816	5	5.03–6.75	8.4–12.5
	2016/2017	*qFI11-11*	11	85.045	85.045	3	3.94–6.14	6.7–11.4
	2016/2017	*qFI11-12*	11	85.078	85.402	24	3.06–10.14	5.2–19.1
	2016/2017	*qFI11-13*	11	85.433	85.739	19	4.28–9.50	7.3–17.1
	2016/2017	*qFI11-14*	11	85.765	86.02	22	3.55–10.35	6.0–19.0
	2016/2017	*qFI11-15*	11	86.174	87.296	97	3.22–11.07	6.5–19.7
	2016/2017	*qFI11-16*	11	87.307	87.332	11	3.89–6.65	6.6–12.3
	2016/2017	*qFI11-17*	11	87.341	87.453	18	4.31–10.09	7.3–19.2
	2016/2017	*qFI11-18*	11	87.689	90.945	289	3.39–10.43	5.8–19.6
LW	2015/2017	*qLW11*	11	38.638	43.749	1	2.50–3.07	4.8–6.2
	2016/2017	*qLW5*	5	68.041	76.614	7	2.60–3.53	4.8–6.4
LL	/	*/*	/	/	/	/	/	/
LI	2015/2017	*qLI2*	2	79.739	79.739	1	2.74–4.43	5.2–7.8
	2015/2016	*qLI8-1*	8	137.077	137.856	2	2.75–3.06	5.4–5.6
	2015/2017	*qLI8-2*	8	120.236	125.565	7	2.62–2.99	4.6–6.2
	2015/2016	*qLI8-3*	8	137.077	137.856	2	2.75–3.06	5.4–5.6
	2016/2017	*qLI10-1*	10	4.143	4.143	1	3.18–4.143	5.4–5.6
	2015/2016/2017	*qLI10-2*	10	93.095	97.12	4	2.50–3.39	5.0–6.6
	2015/2017	*qLI11-1*	11	22.712	23.747	1	2.64–3.55	5.2–7.5
	2015/2016/2017	*qLI11-2*	11	84.488	86.911	20	2.50–3.55	4.6–8.2
	2015/2017	*qLI12-1*	12	72.131	72.523	4	2.84–5.26	4.9–9.3
	2015/2017	*qLI12-2*	12	76.491	77.557	13	2.63–5.52	4.5–9.3
	2015/2017	*qLI12-3*	12	90.176	91.375	6	2.50–4.51	4.3–8.1
	2015/2017	*qLI12-4*	12	103.513	103.513	1	2.53–2.79	4.6–4.8

QTLs responsible for all traits except LL were detected and distributed on LG 1, 2, 3, 4, 5, 8, 10,11, and 12. Most QTLs were located on LG11, responsible for FL, FI, LW, and LI. The number of markers supporting corresponding QTLs was between 1 and 289, accounting for 4.3∼30.9% of the variation in their corresponding traits. The minimum LOD value of all QTLs was 2.5, while the maximum was 17.24.

Specifically, among the fruit-related traits, we detected seven QTLs for FW traits distributed on LG1, 2, 8 and 10. All proportion of variance explained (PVE) values were less than 10% and the QTL with the largest number of supporting markers (seven) was *qFW10-3*. QTLs controlling FL were mapped to LG10 and 11. The region of *qFL10* coincided with *qFW10-1*, indicating they were the same QTL. The remaining QTLs were located on LG11, of which the LOD value of *qFL11-3* was up to 17.24 with three supporting markers, and the corresponding PVE value was as high as 30.9%. There were four QTLs corresponding to FD, which spanned LG8, 10 and 12. On LG10, there were two QTLs (*qFD1-1* and *qFD1-2*) with a total of eight supporting markers. The PVE of *qFD8* on LG8 exceeded 10% (10.7%). Among all leaf and fruit related traits, FI was associated with the largest number of QTLs with a total of 24 distributed on LG1, 3, 4 and 11. Most QTLs (18) for FI were located on LG11 spanning the interval 73.492–90.945 cM (17.45 cM). The number of supporting markers was up to 289. The highest LOD and PVE value belonged to *qFI11-15*, reaching 11.07 and 19.7, respectively. These QTLs on LG11 were gathered tightly with an average interval of less than 1 cM per QTL, suggesting that it might be a cluster region controlling FI collectively.

As for leaf related traits, two stable QTLs were detected for LW, distributed on LG5 and 11, of which five markers supported *qLW5* and the LOD value was up to 3.53. A total of 13 stable QTLs for LI were distributed on LG2, 8, 10, 11, and 12. Of particular note, amongst all 55 QTLs, *qLI10-2* and *qLI11-2* were detectable for 3 years and the interval was 93.095–97.120 cM with four supporting markers and 84.490–86.911 cM with 20 supporting markers, respectively. The PVE of all leaf related traits was within 10%, suggesting their minor-polygene genetic mechanism.

## Discussion

For species without a reference genome, restriction enzyme digestion-based sequencing (Matsumura et al., 2014; Pootakham et al., 2015; Liu L. et al., 2018) or transcriptome sequencing (Li and Jiang, 2014; Zhou et al., 2016; Li et al., 2019) have become commonly used techniques for genome-wide *de novo* SNP discovery. SLAF-seq is an effective restriction enzyme digestion-based sequencing method for developing SNP markers and has been used in many different organisms (Luo et al., 2016; Chang et al., 2018; Fang et al., 2018). In this paper, we used SLAF-seq sequencing to develop a large number of SNP markers for two wolfberry accessions. At the same time, in order to guarantee the quality of the markers, we employed stricter filtering criteria (integrity ≥75%, SD ≥ 0.01) than other researchers (Wang et al., 2012; Zhang et al., 2016; Zhao et al., 2016; Zheng et al., 2018) and successfully generated a high-density genetic map harboring 6,733 SNP markers. This is the first high-density genetic map of wolfberry, which will lay a robust foundation for subsequent QTL mapping studies in order to shed light on the molecular biology of this crop. The map will be a useful tool for the comparative genomics (Swaminathan et al., 2012) and will assist with assembling a chromosome-scale reference genome for wolfberry.

Unlike densely planted crops that are easy to phenotype, F_1_ populations are normally developed for forest trees or shrubs with difficult field-management requirements. In general, the numbers of F_1_ individuals tend to be between 100 and 200 (Wang et al., 2012; Zhu et al., 2015; ıpek et al., 2016; Luo et al., 2016; Zhang et al., 2016; Lu et al., 2018; Zheng et al., 2018). In the present paper, 302 F_1_ individuals were used and the average genetic distance on the integrated genetic map was 0.253 cM, which is a higher resolution than previously reported (0.32–1.16 cM) using fewer individuals (Wang et al., 2012; Zhu et al., 2015; ıpek et al., 2016; Luo et al., 2016; Zhang et al., 2016; Lu et al., 2018; Zheng et al., 2018). This comparison supports the idea that more individuals can bring more recombination exchanges (De Massy, 2013), thus enabling higher resolution, which can improve the accuracy of QTL positioning and accelerate the fine positioning process (Pan et al., 2017; Zhang et al., 2018). Therefore, within an acceptable range, we encourage a population with more offspring to construct genetic maps and map QTLs for woody plants.

We identified a large number of QTLs, of which 55 could be stably detected (Table 6 and Supplementary Figure S2). Among these stable QTLs, *qFL11-3* was detected in samples from 2016 and 2017 with LOD and PVE values up to 17.24 and 30.9%, respectively. *qLI10-2* and *qLI11-2* were mapped from 2015 to 2017 with four and 20 supporting SNPs, respectively, indicating their high reliability. In future research, these SNPs can be developed into KASP or HRM markers (Chang et al., 2018; Liu et al., 2019) to be used for molecular marker-assisted selection after the linkage states between SNP markers and their corresponding traits are verified in additional populations. Moreover, we can further fine map the QTLs by narrowing the region. Backcrossing is a traditional fine mapping strategy for introducing more recombination (Guo et al., 2018). In 2017, we selected F_1_ individuals with appropriate traits and crossed them with the parent Chinese gouqi. From this cross, we have obtained BC_1_ populations and will conduct phenotyping in 2018. In order to save time, we can generate two or more populations simultaneously by choosing an accession containing valuable QTLs that we want to isolate and two other accessions with relative characteristics to hybridize. The populations are used to perform traditional QTL positioning, then develop markers within QTLs and continue to narrow the interval. Grape *VviAGL11* gene mapping was carried out using this method initially locating QTLs to a 1.70 Mb interval and then continuing to shrink the region to 323 Kb, achieving fine positioning of traits for seedlessness (Royo et al., 2018). At the same time, a transcriptome analysis study on the expression patterns among the developmental stages of leaves and fruit will accelerate the candidate gene mining progress (Wang et al., 2017).

QTLs for leaf and grain traits have been mapped or fine mapped in a number of crops (Digel et al., 2016; Tang et al., 2018; Zhai et al., 2018). In wolfberry, we wanted to assess the possibility of finding potential candidate genes through analysis of genome synteny with other species. It has been reported that eggplant, tomato and pepper show a high level of genome synteny (Rinaldi et al., 2016). These species, along with wolfberry, are all members of the Solanaceae and have genomes of 12 chromosomes. Therefore, recent reports of QTLs for leaf size and FW in tomato and pepper enabled us to go forward with the study of genome synteny with wolfberry (Illa-Berenguer et al., 2015; Chunthawodtiporn et al., 2018). We selected *qLI10-2* and *qLI11-2* for leaf related traits and *qFL11-2*, *qFL11-3*, *qFL11-4*, *qFL11-5*, *qFI11-2* and *qFI11-7* for fruit related traits, before extracting the corresponding SNP markers to map with the reference genomes of tomato^1^ and pepper^2^ using a BLAST search^3^ with identity = 90% (Supplementary Table S3). The mapping results showed that almost all SNP markers could be mapped onto the reference genomes of pepper or tomato, suggesting that broad genome fragments were shared between these Solanaceae species. However, we observed that most of the SNP markers in the same QTL region of wolfberry were scattered across different chromosomes, whether comparing with the pepper or tomato reference genomes indicating that a high degree of genome differentiation existed between wolfberry and these two species (Supplementary Table S3). Therefore, we believe that further evaluation is needed to explore candidate genes and underlying QTLs for wolfberry leaf and fruit morphological traits using genome synteny.

In the absence of a reference genome, it usually takes a considerable length of time to clone a gene by map-based cloning (Frary et al., 2000), Chromosome landing is a traditional paradigm to clone QTLs, although polymorphic markers are sometimes not easy to obtain (Tanksley et al., 1995). The *MYB114* gene controlling red skin in pear was identified by anchoring the QTL region of two LOD peak markers to a physical map (Yao et al., 2017) and this method could be used for wolfberry, if a suitable reference genome were available. In recent years, especially with the development of long-read sequencing technologies such as Pacific Biosciences and Oxford Nanopore Technology, *de novo* assembly of a high-quality genome has become easier to realize (http://www.plabipd.de/index.ep). A reference genome of wolfberry will greatly accelerate the cloning of our stably expressed QTLs.

## Conclusion

Our results indicate that SLAF-seq was an effective method to develop high quality SNPs that can be used to construct an ultra-dense genetic map for wolfberry (*Lycium* Linn.) without available reference genome sequences. The genetic map, including 6733 high-quality SNP markers, was constructed using an F_1_ population derived from a cross between “Ningqi NO.1” and “Chinese gouqi”. On account of this high-density genetic map and a set of three continuous years’ phenotyping data, 55 reproducible QTLs were detected as being responsible for leaf- and fruit-related traits. The SNPs containing favorable alleles within the QTL regions could offer promising selective markers for marker-assisted breeding at seedling stage. Our results provide a new pathway to the study of wolfberry molecular genetics and MAS breeding.

## Author Contributions

JZ and YC conceived and designed the experiments. HL, YY, WA, YL, YW, YF, and RW performed the experiments and collected the data. JZ, YX, and XG analyzed and uploaded the data. JZ wrote the manuscript. YX revised the manuscript and edited the language. All authors revised the final submission of the manuscript.

## Conflict of Interest Statement

YX and XG were employed by the company Biomarker Technology Corporation. The remaining authors declare that the research was conducted in the absence of any commercial or financial relationships that could be constructed as a potential conflict of interest.

## Footnotes

1 ftp://ftp.solgenomics.net/tomato_genome/assembly/build_2.50/

2 www.pnas.org/lookup/suppl/doi:10.1073/pnas.1400975111/-/DCSupplemental

3 https://blast.ncbi.nlm.nih.gov/Blast.cgi

## Supplementary Material

FIGURE S1 The frequency distribution histograms of traits in year 2015, 2016, and 2017.

FIGURE S2 Integrated QTLs in the genetic maps.

TABLE S1 Basic sequencing information statistics of all materials.

TABLE S2 Detail information of the integrated map.

TABLE S3 The BLAST results of SNP makers in QTLs for leaf and fruit traits of wolfberry.
