# Supplementary material for: A SNP-Based High-Density Genetic Map of Leaf and Fruit Related Quantitative Trait Loci in Wolfberry (Lycium Linn.)
Source: Front Plant Sci. 2019 Aug 7;10:977. doi: 10.3389/fpls.2019.00977 (PMC6693522; doi:10.3389/fpls.2019.00977)
Supplement: FIGURE S1 — The frequency distribution histograms of traits in year 2015, 2016, and 2017. [file Image_1.pdf]

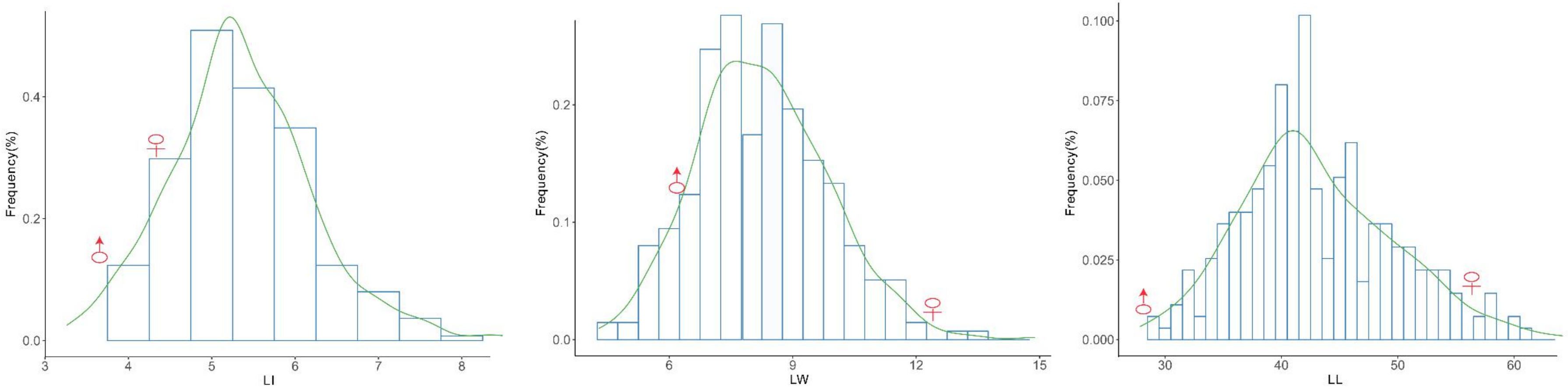

Frequency distribution histograms of Leaf Index (LI), Leaf Width (LW) and Leaf Length (LL) in 2015.

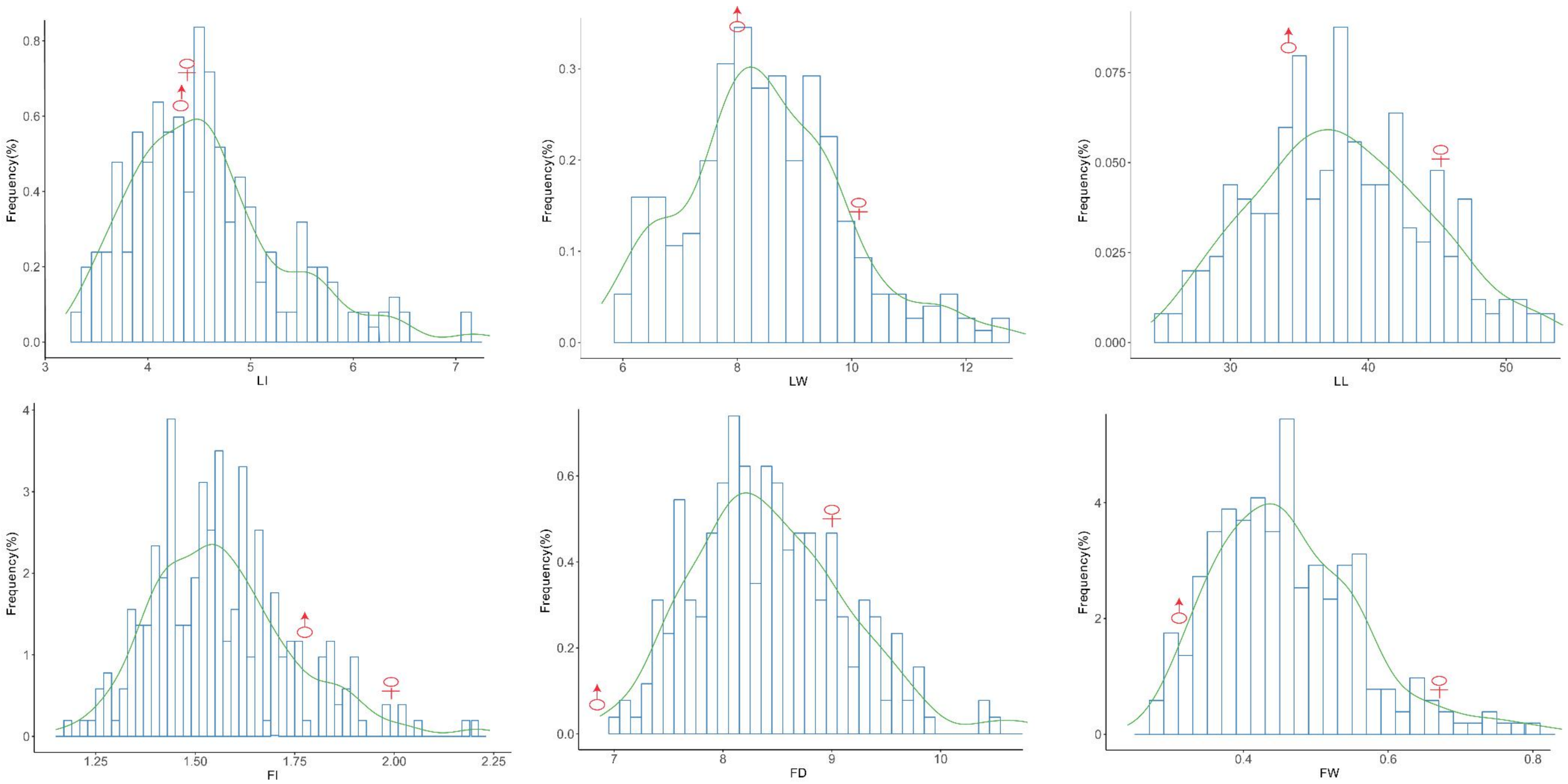

Frequency(%)
LI
3
4
5
6
7
0.0
0.2
0.4
0.6
0.8
Frequency(%)
LW
6
8
10
12
0.0
0.1
0.2
0.3
Frequency(%)
LL
30
40
50
0.000
0.025
0.050
0.075
Frequency(%)
FI
1.25
1.50
1.75
2.00
2.25
0
1
2
3
4
Frequency(%)
FD
7
8
9
10
0.0
0.2
0.4
0.6
Frequency(%)
FW
0.4
0.6
0.8
0
2
4
♂
♀

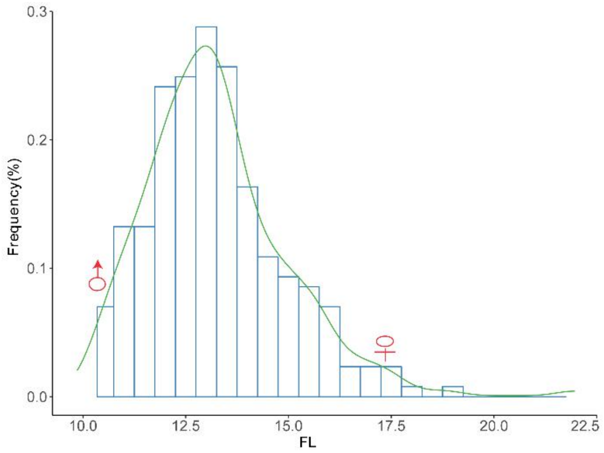

Frequency distribution histograms of leaf index (LI), leaf width (LW), leaf length (LL), single fruit weight (FW), fruit longitude (FL), fruit diameter (FD) and fruit index (FI) in 2016.

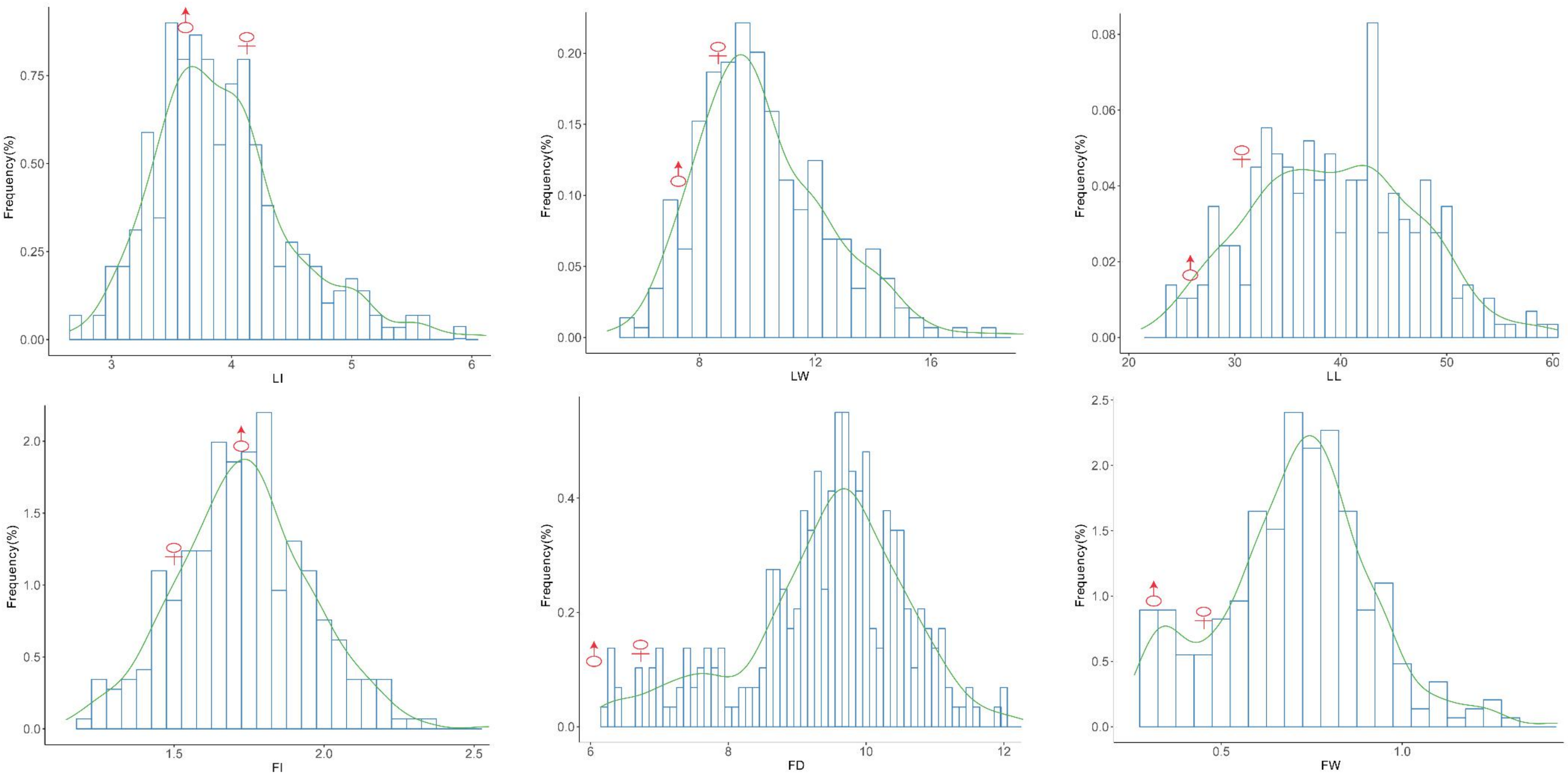

Frequency(%)
LI
LW
LL
FI
FD
FW

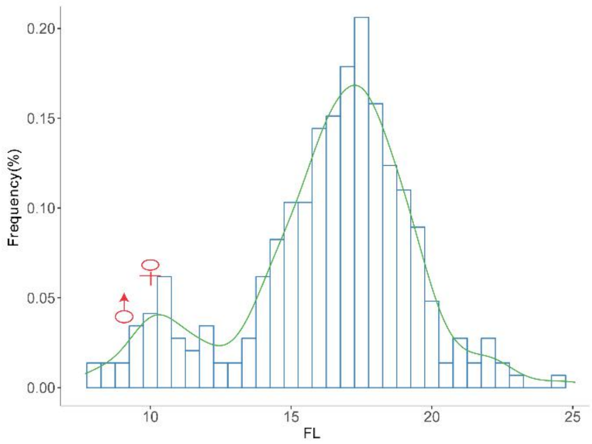

Frequency distribution histograms of leaf index (LI), leaf width (LW), leaf length (LL), single fruit weight (FW), fruit longitude (FL), fruit diameter (FD) and fruit index (FI) in 2017.
