# Supplementary figures and images for: A SNP-Based High-Density Genetic Map of Leaf and Fruit Related Quantitative Trait Loci in Wolfberry (Lycium Linn.)
Source: Front Plant Sci. 2019 Aug 7;10:977. doi: 10.3389/fpls.2019.00977 (PMC6693522; doi:10.3389/fpls.2019.00977)

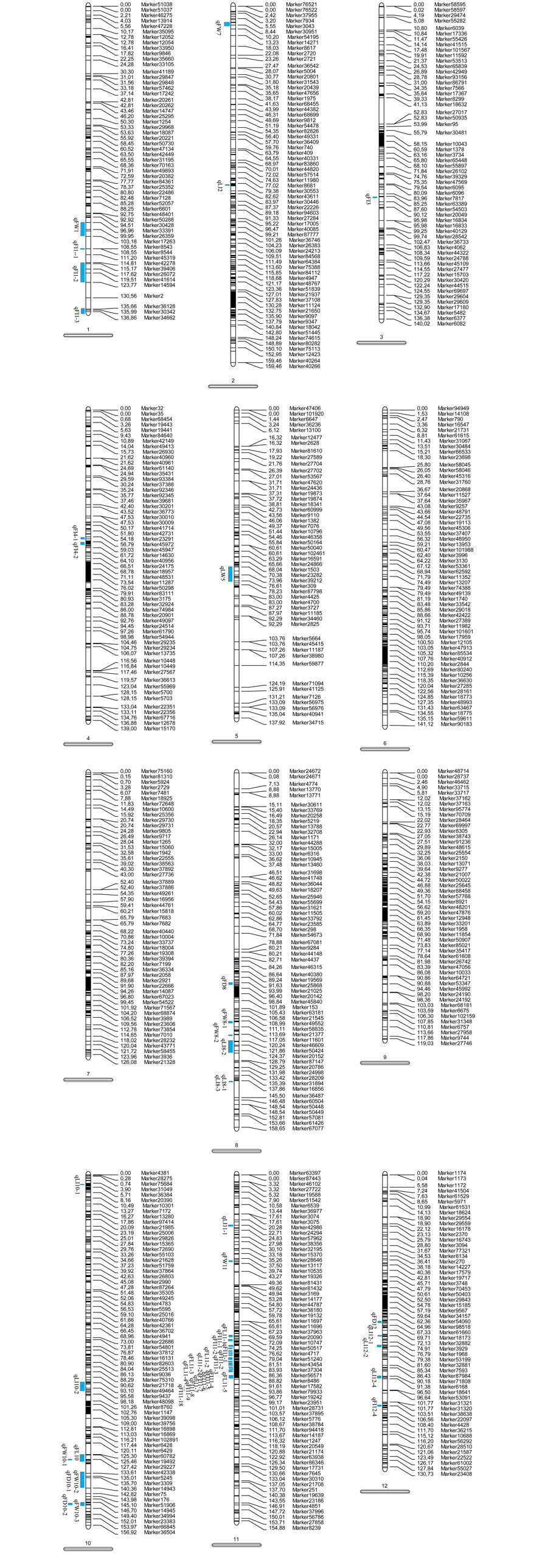

Supplement: FIGURE S2 — Integrated QTLs in the genetic maps. [file Image_2.png]
